# Reduced Let-7a Is Associated with Chemoresistance in Primary Breast Cancer

**DOI:** 10.1371/journal.pone.0133643

**Published:** 2015-07-28

**Authors:** Jiannan Wu, Shunrong Li, Weijuan Jia, Heran Deng, Kai Chen, Liling Zhu, Fengyan Yu, Fengxi Su

**Affiliations:** 1 Guangdong Provincial Key Laboratory of Malignant Tumor Epigenetics and Gene Regulation, Sun Yat-Sen Memorial Hospital, Sun Yat-Sen University, Guangzhou, 510120, China; 2 Depart of Breast Surgery, Sun Yat-Sen Memorial Hospital, Sun-Yat-Sen University, Guangzhou, 510120, China; University of Torino, ITALY

## Abstract

Chemotherapy resistance remains an important problem in the breast cancer clinic. The ability to predict the patients who would respond to a distinct therapy would help to optimize tailored treatment options. miRNAs can mediate a number of genes in response to drug-induced acute cellular stress. Several studies suggest that let-7 miRNA may be involved in the chemosensitivity of cancer cell lines in vitro. However, it is not known whether this phenomenon occurs in clinical breast tumors. The present study showed that lower let-7a expression was associated with epirubicin resistance in primary breast tumors. Moreover, upregulation of let-7a expression sensitized resistant breast tumor cell lines to epirubicin by enhancing cellular apoptosis in vitro. Collectively, these findings indicate that lower expression of let-7a miRNA can induce chemoresistance in breast cancer by enhancing cellular apoptosis and suggest that let-7a may be used as a therapeutic target to modulate epirubicin-based chemotherapy resistance.

## Introduction

Breast cancer is the most prevalent form of cancer in women worldwide, accounting for 23% of all female cancers [1]. Due to the lack of obvious early symptoms, many patients are diagnosed with the disease at late stages. Neoadjuvant chemotherapy is the standard treatment for locally advanced breast cancer, which aims to improve the surgical options, achieve survival from disease and gain information on the tumor response [2]. Although neoadjuvant chemotherapy improves patient survival, the disease outcomes remain dismal for those who do not achieve a complete pathological response after neoadjuvant chemotherapy [3–6]. Anthracycline-based neoadjuvant chemotherapy (doxorubicin or epirubicin) plays an important role in the neoadjuvant chemotherapy treatment of breast cancer. Apparently, not all of the patients benefit from the anthracycline–based neoadjuvant chemotherapy. Thus, identifying the patients who can or cannot benefit from the anthracycline-based chemotherapy will have significant clinical implications in reducing the treatment cost and minimizing drug toxicity.

MicroRNAs (miRNAs) are small, non-protein coding RNAs that play a crucial role in the post-transcriptional regulation of various physiological and pathological pathways such as cell differentiation, proliferation, and apoptosis. Recently, several lines of evidence revealed that miRNAs may influence the patients’ response to chemotherapy [7–9]. The role of the let-7 family of miRNAs in oncogene regulation has been extensively studied. Our previous study demonstrated that let-7 regulates the self-renewal and tumorigenicity of breast tumor-initiating cells; let-7 is rarely expressed in the breast tumor-initiating cells but is expressed in differentiated breast tumor cells [10]. Because the tumor-initiating cells are relatively more chemo-resistant than the differentiated tumor cells, we hypothesized that let-7 may be expressed at relatively lower levels in chemo-resistant breast cancer tissues and that the acquisition of chemoresistance by the breast cancer cells may be modulated by changing the let-7 levels.

In the present study, we examined whether we can predict the response of breast cancer patients to epirubicin-based chemotherapy before treatment using biopsy specimens. The results showed that low let-7a expression levels before chemotherapy is associated with reduced sensitivity of the breast cancer to epirubicin-based chemotherapy. An in vitro study using breast cancer cell lines further confirmed that let-7a is involved in the breast cancer cells’ epirubicin response.

## Materials and Methods

### 1.1. Patients and tissue samples

Formalin-fixed and paraffin-embedded (FFPE) tissues were collected from 39 breast cancer patients before neoadjuvant chemotherapy, from January 2005 to December 2006. Expression of let-7a was evaluated on tissue sections by miRNA in situ hybridization (ISH). Quantitative real-time PCR was performed on tissue sections obtained from snap frozen pre-treatment core needle biopsies of 31 additional patients of the Breast Tumor Center, Sun-Yat-Sen Memorial Hospital, Sun-Yat-Sen University, from January 2008 to December 2009. All patients were affected by a ductal invasive breast cancer. All of the patients received epirubicin-based neoadjuvant chemotherapy according to the NCCN guideline every 21 days for a total of four cycles. This retrospective study has been approved by the ethics boards of Sun-Yat-Sen Memorial Hospital, and the need for written informed consent from the patient was waived by the ethics committee. The patient tissue and data were anonymized throughout the study. Following completion of the neoadjuvant chemotherapy, the patients underwent mastectomy or conserving breast surgery 4 weeks after the last cycle of chemotherapy. The chemotherapeutic response was clinically evaluated by measuring the change in the tumor size in the breast as follows: (a) complete response (CR), disappearance of all known disease; (b) partial response (PR), ≥30% decrease in tumor size; (c) stable disease (SD), <30% decrease or <20% increase in tumor size; and (d) progressive disease (PD), ≥20% increase in tumor size or the appearance of new lesions. In this study, CR and PR were defined as responders, and SD and PD were defined as non-responders.

### 1.2. Detection of miRNAs by ISH in FFPE sections

In situ hybridization detection of let-7a miRNA expression was performed on formalin-fixed paraffin-embedded tissue sections. Briefly, after dewaxing and rehydration, the samples were digested with proteinase K, fixed again in 4% paraformaldehyde, hybridized with the 5’ digoxin-labeled LNA-modified let-7a probe (Exiqon, Woburn, MA, USA) at 49.5°C overnight, and then incubated overnight at 4°C with an anti-digoxin monoclonal antibody (Roche Applied Science, Indianapolis, IN, USA). After incubation in the nitro blue tetrazolium/5-bromo-4-chloro-3-indolyl phosphate staining solution in the dark, the sections were mounted and observed. For the negative control, the hybridization solution was replaced with the pre-hybridization solution. Each section was scored by comparing the cytoplasmic staining intensity of the tumor cells present in each section using a scale of 0–3, with 3 being the most intense let-7a staining.

### 1.3. Cell cultures and treatment

The SKBR3 cell line was obtained from the American Type Culture Collection, and the SK-3rd sphere cell line was generated from the SKBR3 cell line as follows: mice injected with SKBR3 cells in the mammary fat pad were treated with epirubicin weekly for 10–12 weeks until the xenografts reached ~2 cm diameter. The cells from the third passage (SK-3rd) were called the SK-3rd cells and were cultured in suspension to generate mammospheres (SK-3rd sphere cells), which is a type of breast tumor-initiating cell that is epirubicin-resistant and rarely expresses let-7 [10]. The SKBR3 cell lines were cultured in RPMI 1640 (Invitrogen, Carlsbad, CA, USA) supplemented with 10% fetal bovine serum. The SK-3rd spheres were maintained by the mammosphere culture method, as previously described [10], with or without epirubicin. Transfection of the cells with miRNA mimics (Genepharma, Shanghai, China) was performed using Lipofectamine (Invitrogen) as described [2].

### 1.4. RNA isolation and real-time quantitative reverse transcription PCR assay

The total RNA was isolated from the surgical specimens and the breast cancer cells. Briefly, the total RNA was harvested using TRIzol (Invitrogen, Carlsbad, CA, USA) and the RNeasy mini kit (Qiagen, Valencia, CA, USA) according to the manufacturer’s instructions. The cDNA was obtained by reverse transcription of the total RNA using a TaqMan Reverse Transcription Kit (Applied Biosystems Inc., Foster City, CA). Standard curves were generated, and the relative amount of target miRNAs was normalized against the U6 snRNA. In particular, the absolute copy number of the mature miRNAs was determined by real-time quantitative reverse transcription PCR (qRT-PCR) using TaqMan Assays-on-Demand primer and probe sets along with the TaqMan Universal PCR master mix (Applied Biosystems Inc., Foster City, CA, USA) for cDNA amplification. Amplification and analysis were performed on the ABI 7900 sequence detection system. The data were analyzed according to the comparative Ct method and were normalized to the U6 expression in each sample. Moreover, the fold expression was normalized against the sample with the lowest expression level.

### 1.5. Western blot

The protein extracts were resolved by 10% SDS-PAGE and transferred to nitrocellulose membranes. The membranes were incubated with specific primary antibodies and subsequently incubated with a peroxidase-conjugated secondary antibody (Bio-Rad, Hercules, CA, USA). The signal was then detected using the chemiluminescent detection system (PIERCE, Rockford, IL, USA).

### 1.6. Measurements of cell viability and apoptosis

Cell viability was measured using the MTT assay. Briefly, the cells were seeded in a 96-well plate for 24 h and then exposed to various concentrations of epirubicin for 36 h. The cells were then incubated in a 0.1 mg/ml solution of 3-(4,5-dimethylthiazol-2-yl)-2,5-diphenyltetrazolium bromide (MTT) at 37°C for 3 h and lysed in dimethyl sulfoxide at room temperature for 30 min. The absorbance in each well was measured at 580 nm using a microplate reader. Cellular apoptosis was measured using flow cytometry. Briefly, the cells were treated with epirubicin (2 μg/mL) for 36 h. The cells were plated at appropriate densities (approximately 2.5×10^4^ cells per well) in 3 mL of medium in 6-well plates (Nalge-Nunc, Rochester, NY, USA). After the desired treatment period, all of the cells were collected. The cells were washed in medium, centrifuged and then resuspended in 2 mL of medium. The cells were counted, and a volume of media containing 1×10^5^ cells was centrifuged to obtain a pellet. After dislodging the pellet, 100 μL of 1× assay buffer and 5 μL of annexin V-fluorescein isothiocyanate (FITC) were added, and the sample was mixed by gentle tapping. After a 20 min incubation at room temperature in the dark, 400 μL of 1× assay buffer and 10 μL of PI (50 μg/mL) were added, and the samples were immediately analyzed using a Beckman Coulter EPICS-XL MCL flow cytometer (Beckman Coulter Inc., Brea, CA, USA). The green fluorescence (FITC) and red fluorescence (PI) were detected by filtration through 530 nm and 585 nm band pass filters, respectively. For each sample, 10,000 events were collected.

## Statistical Analysis

Statistical analysis was performed using SPSS statistics software (SPSS). All results are expressed as means ± SD, and *P* < 0.05 was used for significance. Statistical analysis was performed using one-way analysis of variance, and comparisons between groups were performed by an independent sample t test or Bonferroni’s multiple comparison t test. Spearman order correlations were performed to measure the association between variables.

## Results

### 2.1. Lower expression of let-7a is associated with epirubicin resistance in breast cancer tumors

**Fig 1:**
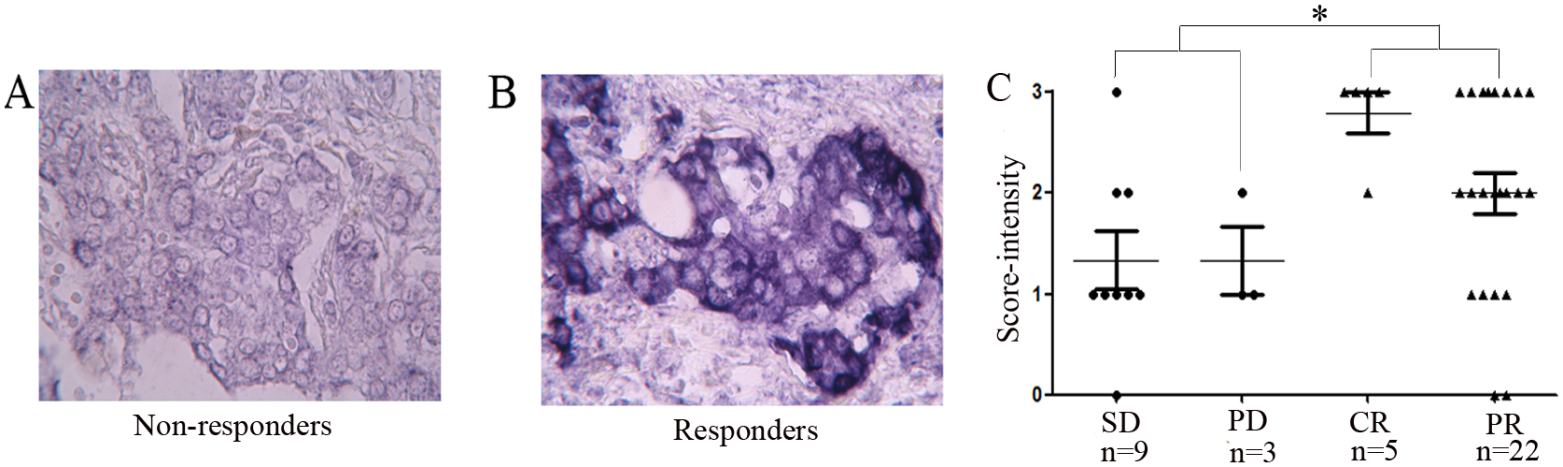
Lower expression of let-7a is associated with epirubicin resistance in breast cancer tumors. Photomicrograph of a primary breast tumor stained for let-7a expression by ISH from patients who (A) failed or (B) responded to epirubicin-based chemotherapy. (C) Scatter plot of the primary breast tumor let-7a ISH scores (range 0–3, with 3 having the highest staining levels) from 27 responders (CR+PR) and 12 non-responders (SD+PD) during epirubicin-based neoadjuvant chemotherapy. Let-7a is expressed at relatively lower levels in the non-responders compared to the responders (*P<0.01).

**Fig 2:**
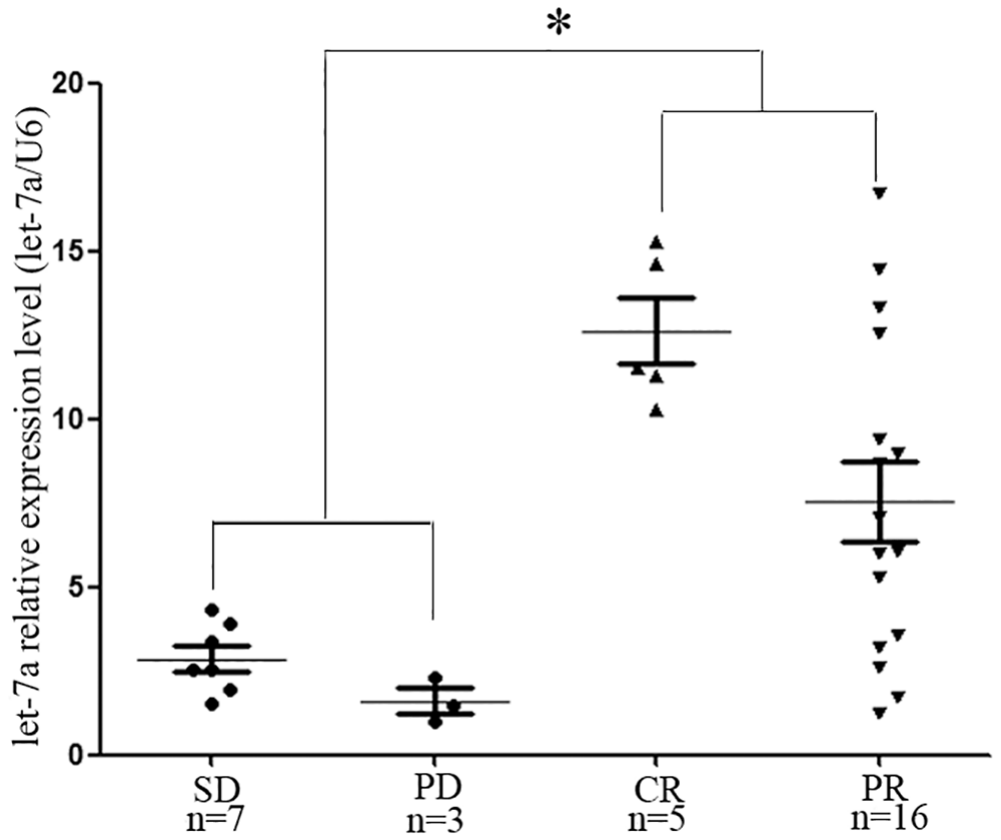
Let-7a expression in 21 responders (CR+PR) and 10 non-responders (SD+PD) by quantitative real-time reverse transcription-PCR. Let-7a is expressed at relatively lower levels in the non-responders (CR+PR) compared to the responders (SD+PD) (*P<0.01). The fold expression was normalized against the sample with the lowest expression level.

To investigate the relationship between the let-7a expression levels and the chemotherapy response, we used DIG-labeled LNA modified miRNA probes to detect let-7a by in situ hybridization in FFPE sections from 39 breast cancer patients who received epirubicin-based neoadjuvant chemotherapy. Two trained pathologists independently scored each tumor on a scale of 0–3, with 3 being the most intense let-7a staining (Fig 1 A and 1B). Among the 39 patients, CR and PR were achieved in 5 and 22 patients, respectively, while SD and PD were observed in 3 and 9 patients, respectively. We found that the scores of the responders were approximately 1.5-fold those of the non-responders (2.14±0.18 vs. 1.333±0.22, P<0.01) (Fig 1C). To validate this phenomenon, we used quantitative real-time PCR to quantitatively detect the expression levels of fresh frozen breast cancer tissues from an additional 31 patients who also received epirubicin-based neoadjuvant chemotherapy. Of the 31 patients, CR and PR were achieved in 5 and 16 patients, respectively, while SD and PD were observed in 3 and 7 patients, respectively. The average level of let-7a expression was 8.76-fold (range 1.21–16.76) in the responders, while the average level in the non-responders was 2.49-fold (range 1.0–4.31), and there was also a significant difference between the two groups (P<0.01) (Fig 2). Taken together, we found that let-7a was expressed at significantly lower levels in the epirubicin-resistant breast tumors. To further investigate the role of let-7 expression in the chemoresistance of breast cancer, we conducted in vitro experiments based on the assumption that let-7a may modulate the chemosensitivity of breast cancer cells.

### 2.2. Let-7a mimics can be effectively transfected into SK-3rd sphere cells and enhance the expression of let-7a miRNA

**Fig 3:**
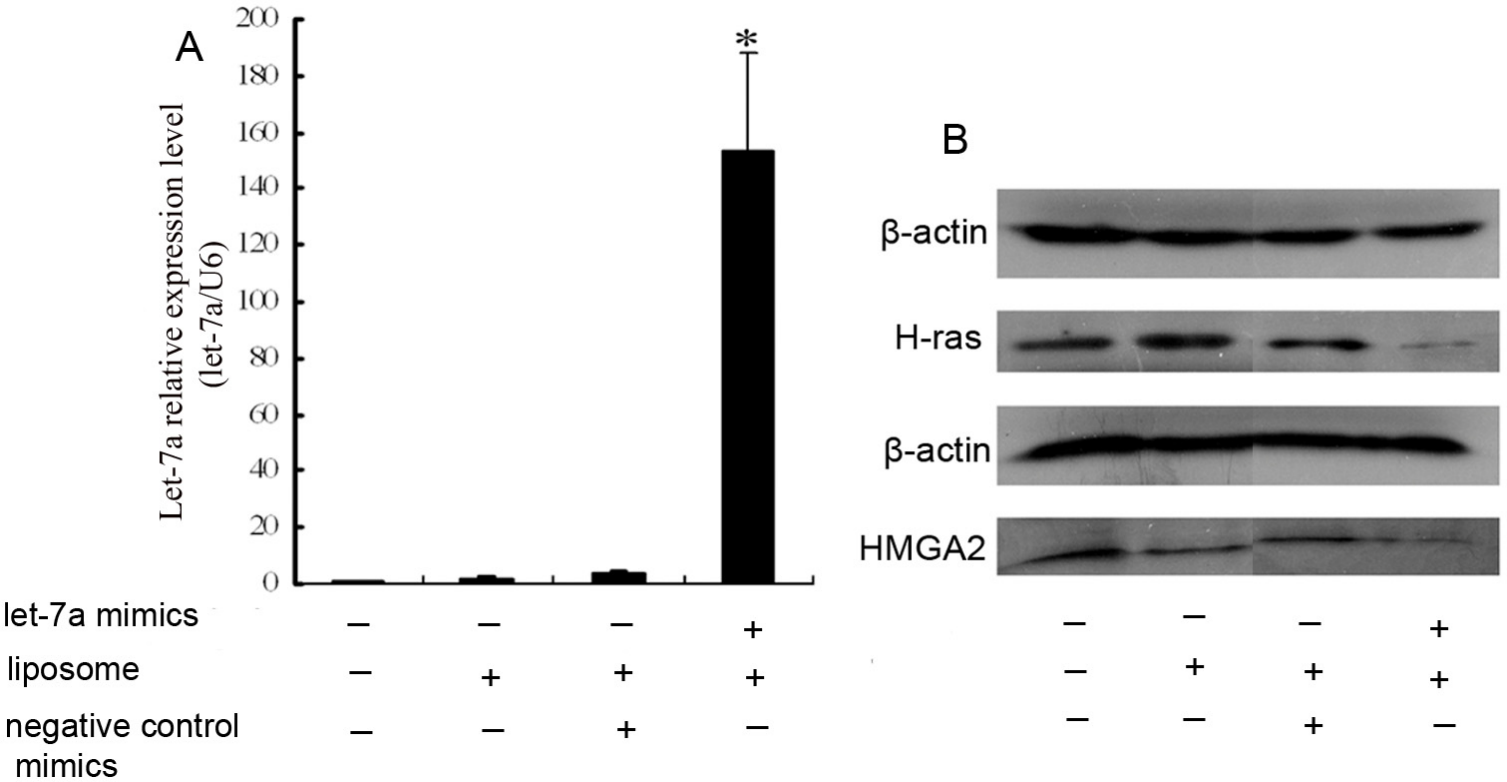
SK-3rd sphere cells were transiently transfected with let-7a mimics, let-7a negative control mimics or liposomes. Transfections with the negative control mimics or liposomes were performed in parallel. (A) The expression levels of let-7a were significantly higher in the SK-3rd sphere cells transfected with both let-7a mimics and liposomes compared to the control groups. The data represent the mean values from three independent experiments. Mean ± SEM. * P<0.01. (B) Let-7a downregulates H-RAS and HMGA2 protein expression in SK-3rd sphere cells. β-actin was used as the loading control. Reduced H-RAS and HMGA2 protein levels were observed in the SK-3rd sphere cells transfected with both let-7a mimics and liposomes compared to the control groups. The experiments were repeated at least three times, and the results were similar to the one shown.

In our previous study, we showed that SK-3rd sphere cells, referred to as breast tumor-initiating cells, rarely expresses let-7 and are resistant to epirubicin, while SKBR3 cells express higher levels of let-7 and are relatively sensitive to epirubicin compared to the SK-3rd sphere cells [10]. Therefore, we focused on the SK-3rd sphere and SKBR3 breast cancer cell lines. Let-7a miRNA mimics were transiently transfected into the SK-3rd sphere cell line to upregulate the expression of let-7a. The effect of the transfection on the expression of let-7a was monitored and validated by quantitative real-time PCR using the QuantiMir RT assay. To control for non-specific effects of the oligonucleotide and liposome, we separately transfected this cell line with an miRNA that lacks homology to sequences of the human genome (negative control miRNA). As shown in Fig 3, let-7a was highly expressed in the SK-3rd sphere cell line cotransfected with the let-7a mimics and liposomes compared to the other control subgroups. As H-RAS and HMGA2 were the targets of let-7 [11, 12], we found that H-RAS and HMGA2 were down-regulated in SK-3rd sphere cells after transfection with let-7a miRNA mimics through Western blot analysis, while there were no effects for the control transfections (Fig 3B). These data demonstrated that let-7a mimics can be effectively transfected into the SK-3rd sphere cell line, resulting in normal let-7a functions.

### 2.3. Induction of let-7a expression restores chemosensitivity and increases apoptosis after epirubicin chemotherapy

**Fig 4:**
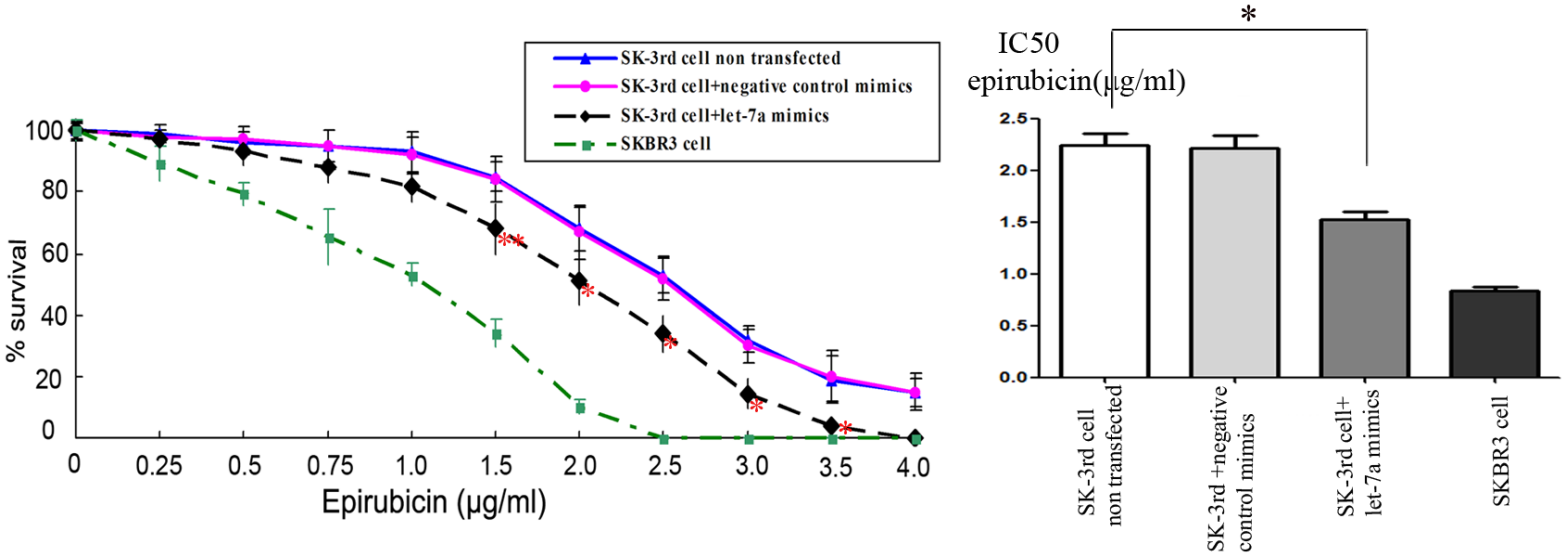
Let-7a modifies the drug sensitivity of SK-3rd sphere cells. The SK-3rd sphere cells were transfected with 25 nM of let-7a mimics or control mimics for 24 h and then exposed to various concentrations of epirubicin for 36 h. The cell survival was then assessed using the MTT assay. The data represent the mean values from three independent experiments. Mean ± SEM, * P<0.01, **P<0.05.

**Fig 5:**
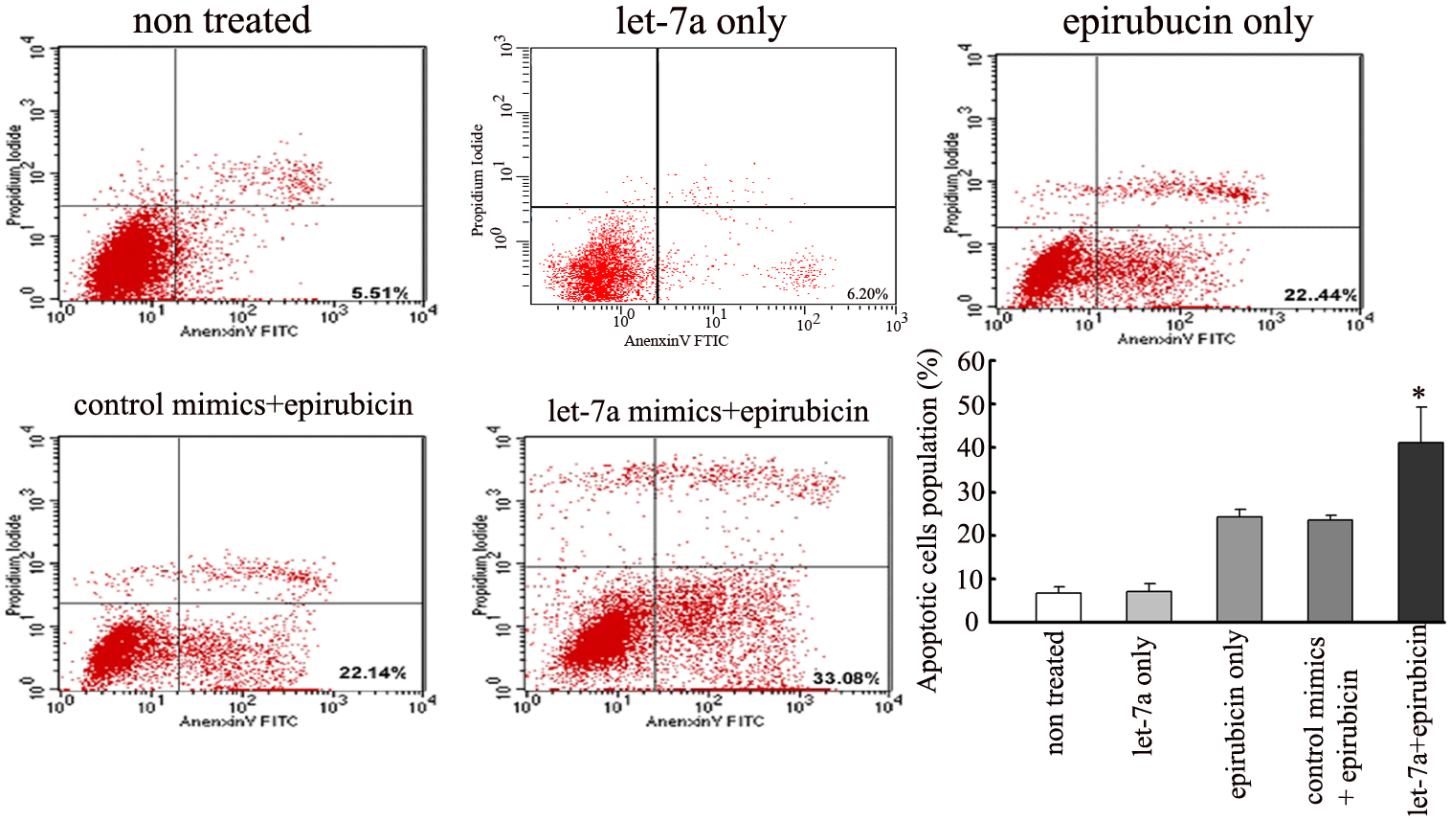
Let-7a enhanced the drug-induced apoptotic cell death in SK-3rd sphere cells. The cells were transfected with 25 nM of the let-7a mimics or control mimics for 24 h and then treated with 2 μg/mL epirubicin for 36 h, followed by the Annexin V binding assay. The experiments were repeated at least three times, and the results were similar to the one shown. The number represents the percentage of cells in the quadrant. Increased apoptotic cell populations were observed in SK-3rd sphere cells transfected with the let-7a mimics compared to the negative control groups. The data represent the mean values from three independent experiments. Mean ± SEM. *P<0.01.

To determine whether modulation of let-7a expression impacts the epirubicin response, we enhanced let-7a expression in the SK-3rd sphere cell line. Using the MTT assay, we found that let-7a upregulation in the epirubicin-resistant SK-3rd sphere cell line sensitized this cell line to epirubicin because the IC50 for epirubicin in the let-7a transfected cells and the non-transfected cells was 1.53±0.07 μg/mL and 2.25±0.11 μg/mL, respectively (P<0.01) (Fig 4). At present, apoptosis inhibition is one of the mechanisms of drug resistance in tumor cells. The externalization of phosphatidylserine is the hallmark of apoptosis. Thus, flow cytometric analysis of cells stained with Annexin V was employed to investigate the drug-induced apoptosis in the SK-3rd sphere cells. Upregulated let-7a expression increased the apoptotic cell population in the SK-3rd sphere cells compared to the negative control groups (Fig 5). Therefore, these results suggest that let-7a may play a role in regulating the chemosensitivity of breast cancer cells to epirubicin treatment by increasing cellular apoptosis.

## Discussion and Conclusions

Chemotherapy resistance remains an important problem in the breast cancer clinic. Currently, epirubicin continues to play a very important role in the neoadjuvant and adjuvant chemotherapy of breast cancer, but some patients do not respond well to this agent. Therefore, the ability to predict the patients who would respond to a distinct therapy would help to optimize tailored treatments. In our previous study, we demonstrated that let-7 can regulate the self-renewal of breast tumor-initiating cells, and let-7 was rarely expressed in breast tumor-initiating cells, which are relatively resistant to epirubicin [10]. Therefore, in the present study, we investigated the relationship between let-7a expression levels and the response of breast cancer specimens to epirubicin to determine whether we could predict the response to epirubicin-based chemotherapy by analyzing let-7a expression in breast cancer using core needle biopsy specimens. The results showed that lower let-7a expression is associated with lower chemosensitivity in breast cancer patients. The results also showed that the let-7a expression mediates the chemosensitivity of breast cancer by increasing cell apoptosis.

Several studies have reported the clinical utility of miRNA expression for the prediction of chemosensitivity. Sugimura et al [13] performed quantitative RT-PCR in 74 patients with esophageal cancer who had received cisplatin-based chemotherapy and reported that let-7b and let-7c were expressed at lower levels in chemotherapy-resistant patients. Another study by Yang et al [14], which evaluated the expression of miRNAs from 69 epithelial ovarian cancer patients who had received cisplatin-based chemotherapy using an miRNA microarray, showed that let-7i expression was significantly higher in the complete response group compared to the incomplete response group. In the present study, we found that let-7a expression was significantly lower in the non-responding chemotherapy patients. Therefore, our current data are consistent with the previous studies, suggesting that let-7a may be used as a biomarker to predict the chemotherapy response in breast cancer.

The role of the let-7 family in chemosensitivity has been examined in several in vitro studies. In esophageal cancer cells, let-7 expression may modulate the chemosensitivity to genotoxic chemotherapy in esophageal cancer cells through the IL-6/STAT3 pathway [13]. In hepatocellular carcinoma cells, let-7 negatively regulates Bcl-xL expression and potentiates sorafenib-induced apoptosis. In melanoma, Serguienko et al [15] found that upregulating let-7a expression in doxorubicin-treated WM234 cells can induce a striking increase in cellular apoptosis. Some findings differ, however. For example, Tsang et al [16] reported that let-7a may suppress the drug-induced apoptosis in A431 and HepG2 cells by targeting caspase-3, a well-known effector caspase in apoptosis. In the present study, we found that upregulating the expression of let-7a can enhance apoptosis and reduce the survival of SK-3rd sphere cells exposed to epirubicin, and let-7a miRNA was subsequently found to enhance the chemosensitivity of SK-3rd sphere cells. Therefore, we are currently generating a better understanding of epirubicin chemoresistance in breast cancer, and the present study suggests that let-7a plays a role as a tumor suppressor in regulating breast cancer chemosensitivity.

Clearly, our approach is not free from limitations. First, the clinical results were based on a retrospective analysis using core needle biopsy specimens obtained from patients who received neoadjuvant chemotherapy, and we had only enrolled only 70 breast cancer tissues due to the limited number of breast cancer patients who received neoadjuvant chemotherapy. Second, we did not investigate the mechanisms of let-7a that are involved in inducing apoptosis in SK-3rd sphere cells. We will investigate it further. Third, before we can apply the findings that let-7 expression can be used in the clinic to predict the response of breast cancer to epirubicin, we need to validate this result in a prospective multicenter clinical trial.

In conclusion, we identified that let-7a is an important mediator of the epirubicin response in both breast cancer tissues and tumor cell lines. Our results suggest that let-7a expression may emerge as an important biomarker for predicting the clinical response to epirubicin and may be used as a therapeutic target to modulate epirubicin-based chemotherapy resistance.
